# Secreted production of assembled Norovirus virus-like particles from *Pichia pastoris*

**DOI:** 10.1186/s12934-014-0134-z

**Published:** 2014-09-10

**Authors:** Jaime Tomé-Amat, Lauren Fleischer, Stephanie A Parker, Cameron L Bardliving, Carl A Batt

**Affiliations:** Department of Food Science, Cornell University, Ithaca, NY 14853 USA

## Abstract

**Background:**

Norovirus *virus-like particles* (NoV VLPs) have recently been explored as potential vaccine platforms due to their ability to produce an effective immune response. Expression of the main structural protein, VP1, leads to formation of self-assembled particles with similar characteristics to the original virus. These NoV VLPs have been expressed in *Escherichia coli*, yeast and insect cells. Expression in *E. coli* and insect cells share downstream processing issues due to the presence of inclusion bodies or the need for numerous purification steps. NoV VLPs have also been produced in the yeast *P. pastoris*; however the protein was only expressed intracellularly.

**Results:**

We have successfully expressed and secreted the VP1 protein in the novel *P. pastoris* strain, *Bg11,* using the methanol inducible pJ912 expression vector, containing the cDNA of NoV VP1. Expression of the VP1 protein in *Bg11* was carried out in a 1.5 L bioreactor resulting in a total yield of NoV VLPs greater than 0.6 g/L. NoV VLPs obtained from the culture supernatant were purified via ion-exchange chromatography, resulting in particles with a purity over 90%. The average size of the particles after purification was 40 nm. Transmission electron microscopy was used to visualize the morphology of the particles and saliva-binding assay confirmed that the NoV VLPs bind to Histo-Blood Group Antigens (HBGA).

**Conclusions:**

In this study we describe the expression and characterization of fully assembled Norovirus *virus-like particles* obtained from *P. pastoris*. The particles are similar in size, morphology and binding capacity, as previously described, for the original NoV. Our results detail the successful expression and secretion of VLPs in *P. pastoris*, improving their candidacy as a vaccine platform.

## Background

NoV VLPs have emerged as a promising candidate for a vaccine platform due to their capacity to elicit a strong humoral and cellular immune response. Several NoV VLPs constructs have been developed through insertion of small antigenic epitopes into the NoV VLPs such as the T cell epitope of murine cytomegalovirus, the CD4+ T cell epitope of murine rotavirus VP6, and the M2 extracellular epitope of influenza virus. Studies of these constructs in mice demonstrated an increase in antibody response and immune system activation [1]. VP1 is the main structural protein of Norovirus, and it consists of 555 amino acids, with a molecular weight of approximately 60 kDa. The VP1 protein includes two distinct structural domains, S for shell and P for protruding. The S domain is comprised of the first 225 amino acids, and is essential for the formation of the icosahedrons. The P domain is further divided into two subdomains, which are involved in dimeric contacts that increase the stability of the capsid [2]. The P2 subdomain is responsible for receptor binding and immune reactivity [3]. X-ray crystallography studies show that 180 subunits of VP1 form a T = 3 icosahedral virion [4]. The expression of VP1 protein leads to the formation of *virus-like particles* by self-assembly and these particles are morphologically and antigenically similar to the infectious virus [5,6].

Due to its particulate nature and repetitive ordered structure, NoV VLPs are highly immunogenic, enhancing immune activation by effector cells [7]. The repetitive antigenic epitopes lead to crosslinking of B cell immunoglobulin receptors and B cell activation [8–10]. NoV VLPs are optimal for dendritic cell uptake, activating innate and adaptive immune responses [11–13]. These features support NoV VLPs as an effective vaccine platform [11,14–16].

Since Noroviruses do not replicate in tissue culture, several expression systems have been studied for NoV VLPs production including *Escherichia coli*, *P. pastoris*, insect cells and transgenic plants [17–20]. Ease of strain development, scalability and product yield are important factors in selecting an expression system. While *E. coli* is a widely preferred system, protein complexity may present obstacles through the inability to perform post-translational modifications in *E. coli* and the formation of inclusion bodies that need to be disrupted to form functional virions [21]. Thus, *P. pastoris* and insect systems are advantageous alternatives to *E. coli* expression [22]. To date, NoV VLPs production in insect cells has resulted in yields of approximately 100 mg/L [23–25] but the NoV VLPs are produced alongside enveloped baculovirus particles requiring more complex purification [26]. Insect cells expression also requires a complex growth medium and cell growth takes 18–24 h per division. *P. pastoris* medium is less complex, and has a significantly faster doubling time (90 min). *P. pastoris* is also easy to maintain, low-cost, easily scalable and has the ability to produce posttranslational modifications [27]. Another advantage of using the *P. pastoris* expression system is its ability to secrete the product into the culture media, resulting in a less complex purification process. The hepatitis B surface antigen was successfully expressed on a VLP platform in *P. pastoris* [28]. However, the VLPs were expressed intracellularly [20,28–31] compromising the final yield and increasing the complexity of the purification process [23,32,33].

In this report we describe, for the first time to date, the secretion of NoV VLPs in *P. pastoris*, with a yield over 0.6 g/L. The VLPs were expressed using controlled methanol induction, in a 1.5 L bioreactor and purified by anion exchange chromatography. These particles have been characterized and fully assembled virions are shown to be present in the medium during fermentation without evidence of cell lysis.

## Results

### Expression and purification

**Figure 1:**
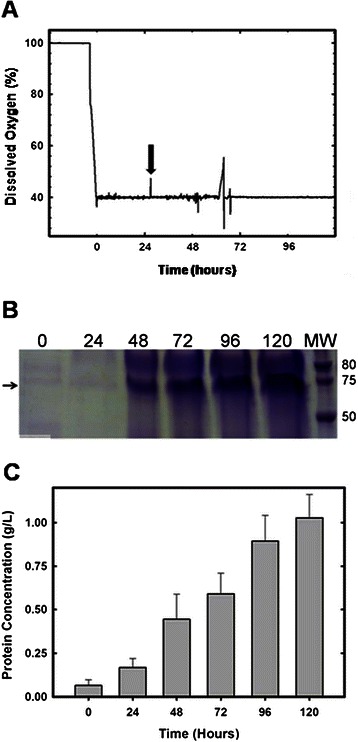
**NoV VLPs production. A)** On-line methanol control performance. Induction phase starts 24 h after inoculation. The arrow indicates beginning of methanol addition. **B)** Every 24 h aliquots were taken from the fermentation media. Cells and supernatant were separated by centrifugation and 10 μl of each supernatant sample was loaded into a SDS-PAGE gel. **C)** Protein concentration was determined for each aliquot, taken every 24 h, by Bradford.

**Figure 2:**
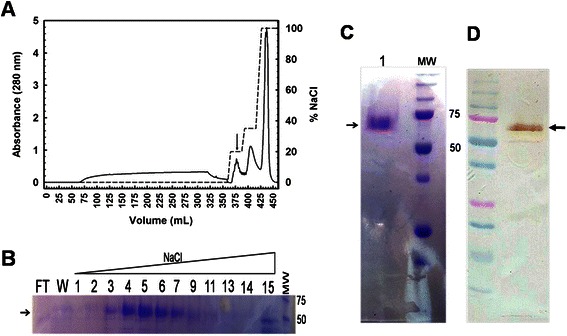
**NoV VLPs purification.** Time course for VLP production. Coomasie brilliant blue stained SDS-PAGE of production and purification of the VP1 protein. **A)** Chromatogram of VLP purification. Solid line represents absorbance at 280 nm and dashed line percentage of NaCl in the buffer. The arrow indicates the elution peak of the VLPs. **B)** Aliquots from ion exchange purification. The numbers correspond to the aliquot number during the NaCl gradient. After purification, pools 4 to 7 where dialyzed against BA 50 mM and lyophilized. Part of the lyophilized protein was resuspended in PBS. **C)** and **D)** SDS-PAGE and Western Blot using anti-NoV from this pool are shown, respectively. In each gel the molecular weights are represented with the correspondent value. Arrows indicate the mobility of the VP1 band in the SDS-PAGE.

*P. pastoris Bg11* cells were electroporated with 10 μg of linearized plasmid containing the secretion α-factor fused with the cDNA encoding the VP1 protein. Eight colonies were selected for further analysis. From these eight colonies, the colony with the highest protein expression, determined by western blot, was selected for a 1.5 L fermentation. A single colony was grown in 100 mL of BMGY as a preinoculum for the fermentation. Fermentation was initially grown in batch phase in medium with glycerol to increase biomass. The end of the batch phase was detected by a DO-spike (Figure 1A), after which protein expression was induced with methanol at a concentration of 2 g/L. Every 24 h an aliquot was taken from the bioreactor and dry cell weight was recorded and the culture supernatant was stored at 4°C. Protein expression was analyzed via SDS-PAGE gel (Figure 1B), and total protein concentration was measured by Bradford (Figure 1C). The final dry cell weight for the fermentation was 300 g/L. After 120 h of methanol induction, the extracellular medium was collected and the NoV VLPs were purified using ion-exchange chromatography, on a Sepharose Q XL column. A step gradient was used to elute the NoV VLPs. The chromatogram showed three major elution peaks (Figure 2), where NoV VLPs elute in the peak between 0.1-0.2 M NaCl, as expected [34,35]. The elution fractions containing the NoV VLPs were pooled together and analyzed. The SDS-PAGE gel showed a band at 62 kDa corresponding to the molecular weight of the VLP protein. Western Blot using the anti-NoV serum also revealed a positive band at 62 kDa confirming the expression of the full-length VP1 protein (Figure 2). Protein concentration was over 1 g/L in the fermentation supernatant, and approximately 600 mg after protein purification.

### Cell lysis

**Figure 3:**
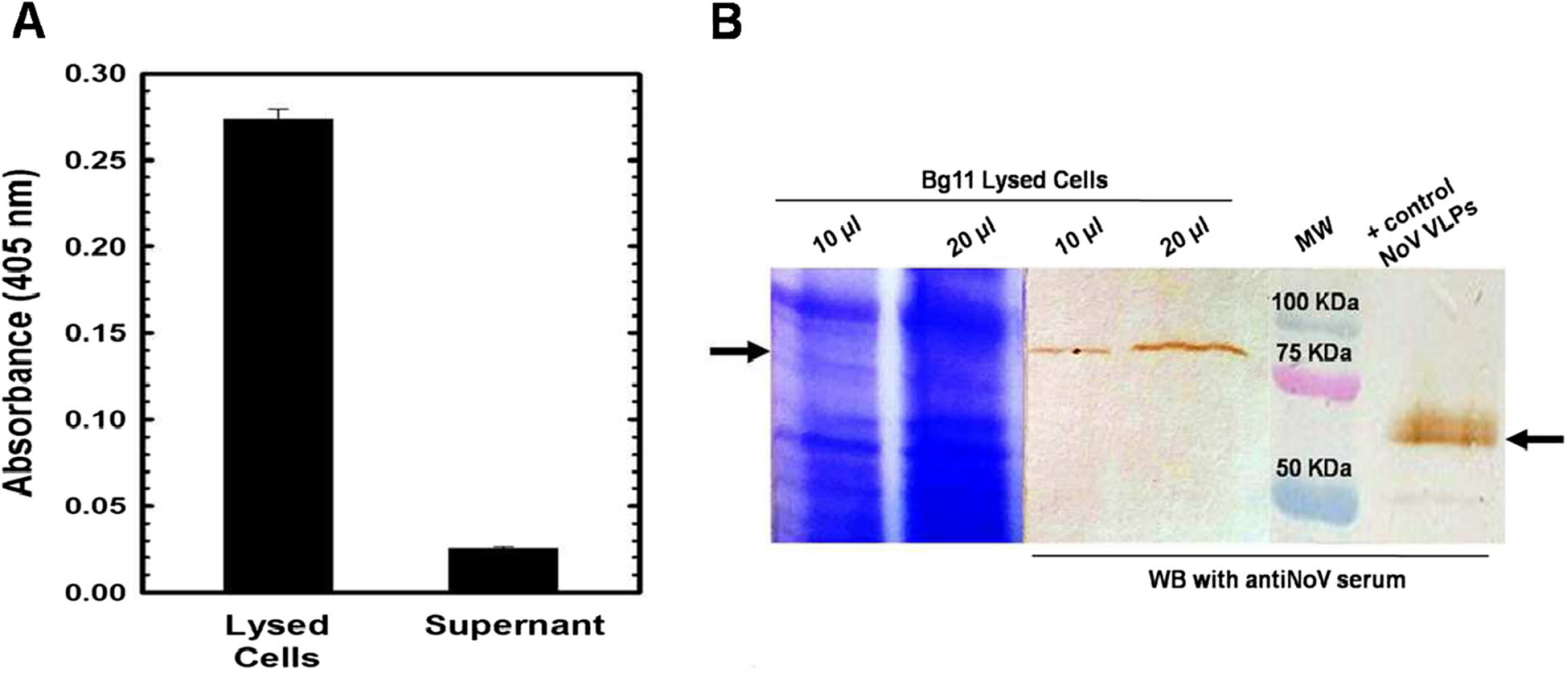
**Cell lysis assay. A)** α-glucosidase detection assay. A sample from the bioreactor was centrifuged and the supernatant was separated from the cells that were lysed. Lysed cells and supernatant were incubated at 37°C with pNPG. The appearance of color (measured as absorbance at 405 nm) is due to the presence of α-glucosidase in the reaction. **B)** SDS-PAGE and Western Blot of culture samples using antiNoV serum. The first four lanes correspond to lysed cells at the end of the fermentation and last lane to a control spiked with NoV VLPs.

The band detected in the Western Blot at 62 kDa with antiNoV serum suggests secretion of the VP1 protein, but to confirm the absence of appreciable cell lysis an intracellular marker, α-glucosidase, was measured in the culture media [36]. A reference curve using lysed cells was constructed to correlate the activity of this enzyme with the volume of lysed cells. After reacting with pNPG, absorbance was measured at 405 nm (Data not shown). Subsequently, a 500 μl sample was taken from the bioreactor, and cells and media were separated by centrifugation. Cells were lysed, and both lysate and media were analyzed. The results were 1.1 ± 0.021 UA for the lysate and 0.07 ± 0.003 UA for the media (Figure 3A). Based upon the activity, the amount of cell lysis was less than 7% of the total cell population. Afterwards, a sample of lysed cells from the fermentation and a sample of the supernatant were used for SDS-PAGE and Western Blot analysis using antiNoV serum. The sizes of the bands observed in the Western Blot were 62 KDa for the supernatant sample and 77 KDa for the lysed cell sample (Figure 3B) suggesting the presence of the α-factor not correctly cleaved in the lysed cells and the correct cleavage of the α-factor in the secreted VP1 protein.

### Saliva-VLP binding assay

**Figure 4:**
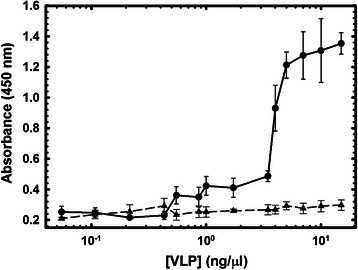
**HBGA-saliva binding assay.** HBGA-binding assay. Plates were coated with A-type saliva, and incubated with different concentrations of VLP (solid line) or culture media after VLPs purification (dashed line). Anti-NoV serum was used for VLP detection. Absorbance values are shown as the mean of six independent experiments.

To confirm the activity of *P. pastoris* expressed NoV VLPs, a purified sample was tested for epitope binding in saliva. NoV VLPs have been shown to recognize and bind epitopes H, A/B, and O-secretor types in saliva samples [33,37]. For this study saliva A-phenotype was used in the binding assay and the results confirmed binding of the VLPs to the saliva samples (Figure 4). The ID_50_ obtained in the ELISA was 0.8 ng/μL.

### Size and morphology characterization by TEM and DLS

**Figure 5:**
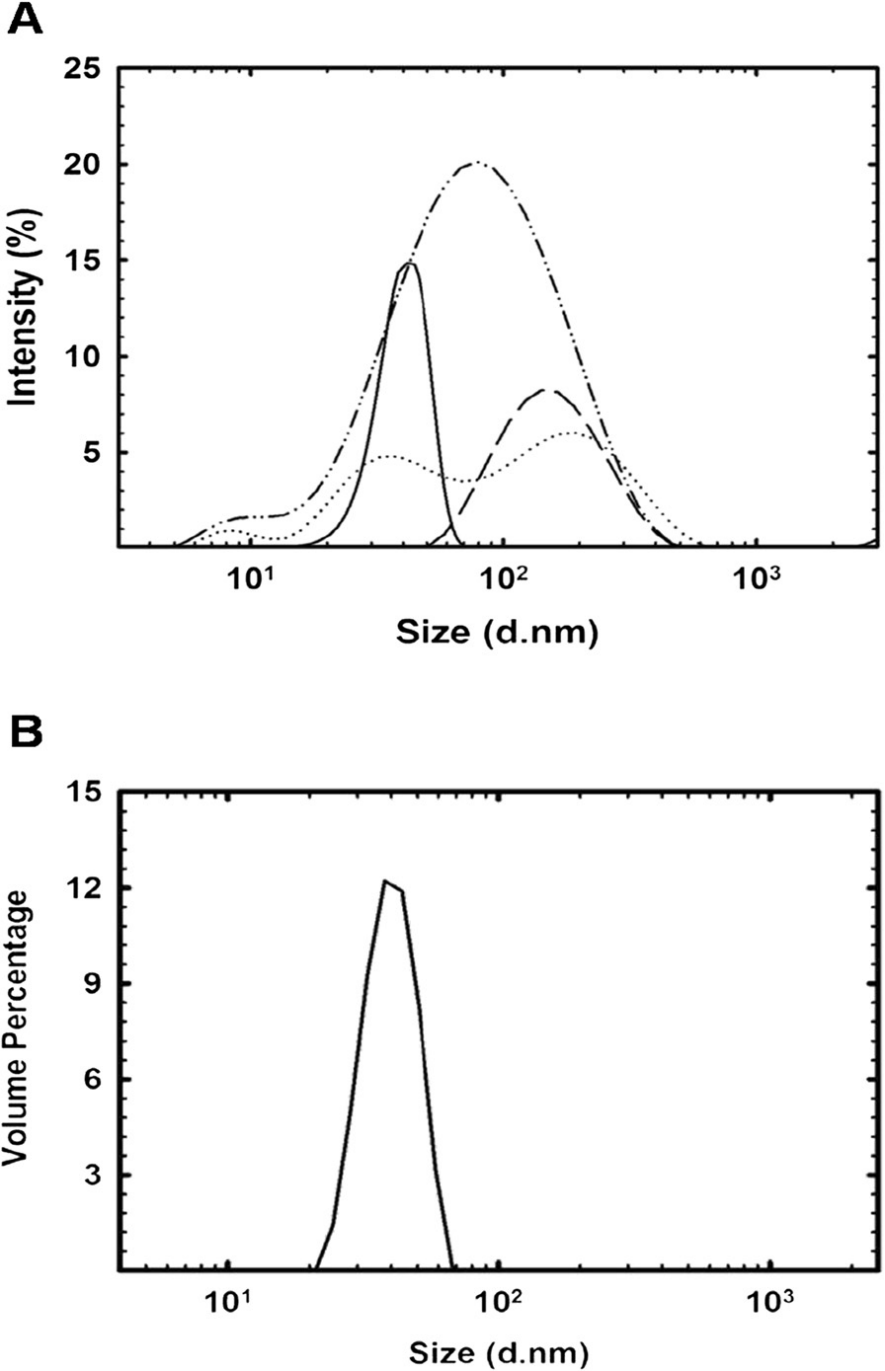
**NoV VLPs dynamic light scattering assay. A)** Particle size distribution samples measured by DLS from the fermentation (dashed line), after ion exchange chromatography (dash-dotted line) and after resuspension of the lyophilized protein (solid line). The dotted line corresponds to the same sample as the solid line does after one week at 4°C. Results were calculated as an average of four measurements. **B)** Particle size distribution of a purified NoV VLPs sample expressed as volume percentage.

**Figure 6:**
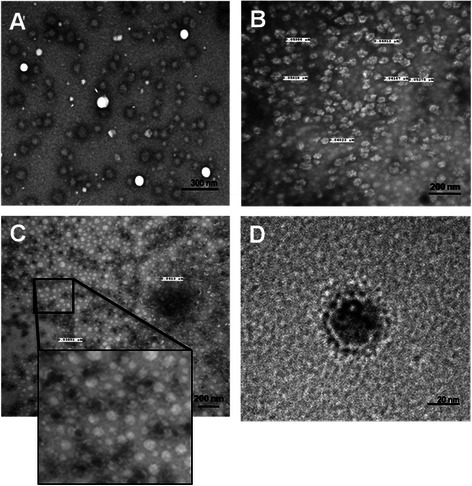
**NoV VLPs transmission electronic microscopy.** Images of VLP obtained using transmission electronic microscopy: **A)** corresponds with a concentrated sample from the culture supernatant during the fermentation, **B)** and **C)** corresponds to two different amplifications of sample after ion-exchange purification and **D)** shows the morphology of a NoV virus like particle.

The particle size and morphology of the *P. pastoris* expressed NoV VLPs were characterized following the fermentation and purification. To determine the average size of the particles during the process, different samples were studied using DLS. Samples from the culture media and purification were concentrated using an Amicon Ultra Membrane (Millipore) for DLS measurements. From the purified pool, an aliquot was dialyzed against 50 mM ammonium bicarbonate pH 6.0 and then lyophilized. Figure 5 shows the average size of the particles during the fermentation (≈107 nm), chromatography (≈70 nm) and resuspended lyophilized product (≈40 nm). Furthermore, the dotted line in Figure 5 depicts the average size of the population after having been stored at 4°C for one week, indicating particle aggregation at low temperatures, as described previously [38]. The particle size and morphology was further examined via transmission electronic microscopy (Figure 6). Figure 6A shows particles present, during the fermentation, in the culture supernatant with a range of sizes (40–100 nm), corresponding with the data obtained by DLS. TEM images display the morphology of the NoV VLPs after chromatography in Figure 6B and C. Figure 6D represents a zoomed-in view of one of the particles after purification

.

## Discussion

In this study we report a method for the production of fully assembled NoV VLPs with high yield from a recombinant expression system. Several expression systems have been studied for this purpose, where insect cells are the preferred system for NoV VLPs expression [11,23,35,38]. The yield reported for this system is on the order of 100 mg/L of culture [23,25]. Yields were increased using *E. coli*, but this system presents drawbacks including lack of post-translational modifications, a deficiency in the formation of disulfide bonds, the formation of insoluble inclusion bodies, as well as the presence of endotoxin, as was observed when parvovirus B19, CCMV and CMV were expressed in *E. coli* [22]. Recently, there have been reports of expression in *E. coli* involving a new kind of NoV particles based on expression of only the P2 domain of the VP1 protein. This protein forms smaller particles, called p-particles. However, the relatively high endotoxin levels in the culture medium present a challenge for clinical trials. These p-particles show similar binding patterns to those of the NoV VLPs when assayed against HBGA groups [23,32,36], but with one order of magnitude less binding than the NoV VLPs [23].

The *P. pastoris* expression system has advantages as it is widely acceptable for pharmaceutical applications and is easily scalable. Currently, there are no reports of NoV VLPs or p-particles obtained from the culture medium of *P. pastoris*. However, intracellular yields on the order of 10 mg/L have been reported [20,30]. Prior studies of NoV VLPs expressed in *P. pastoris* confirm that the particles stimulate systemic and mucosal immunity in mice [31]. Our work is the first to describe the production of fully assembled NoV VLPs obtained directly from the culture medium. Purification of the NoV VLPs was carried out using ion-exchange chromatography [34]. Other studies have compared several systems for VLP purification [11,36,38], such as CsCl and ultracentrifugation, which are well known for achieving high purity levels. However, this method of purification is not easily scaled for larger volumes. Ion-exchange chromatography was selected due to its previous success with NoV VLPs purification, as well as its scalability for large-scale production and purification [39,40].

The NoV VLPs reported here have binding characteristics similar to previously reported NoV VLPs [20,23]. A saliva-binding assay confirmed that the NoV VLPs produced in *P. pastoris* were correctly assembled, as indicated by a binding pattern comparable to earlier reports [38]. The ID_50_ obtained with NoV VLPs after purification is 0.8 ng/μL, in accordance with previous reported values (≈0.3 ng/μL) [23,34]. Additionally, DLS and TEM analysis of particle size and morphology are highly similar to those of previous reports [20,34], and confirmed the presence of NoV VLPs in the fermentation supernatant and after purification by ion-exchange chromatography. The average diameter of the NoV VLPs in the bioreactor (>60 nm) could be explained by the high salt concentration in the media, which has been shown to affect the particle size, as described with HPV VLPs expression in *P. pastoris* [41]. Aggregation of the VLPs could be prevented through lyophilization, the use of detergents, sorbitol, or other buffers [20,41,42].

Further characterization of NoV VLPs will require in vivo studies to examine the immunogenicity of the particles as well as examine their potential use as vaccine platforms. In addition, recent publications, regarding production in yeast, have showed that specific mutations in the α-factor secretion signal enhanced the amount of secreted protein in the extracellular medium [43]. This presents a potential method of increasing production levels of NoV VLPs.

Implementation of NoV VLPs as a vaccine platform requires a scalable and well-characterized expression system for clinical applications. The work presented here shows, for the first time to date, the production of NoV VLPs using *P. pastoris*, obtained from the culture medium, containing a multi-cloning site for antigen insertion, with similar binding and morphological characteristics as NoV VLPs produced in other systems. This work is a step towards the development of NoV VLPs as vaccine platforms.

## Conclusions

We have produced Norovirus *virus-like particles* from *P. pastoris*, using the novel strain, Bg11. The NoV VLPs were produced using a bioreactor and purified directly from the culture medium, via ion-exchange chromatography, with a final purity product over 90%. The NoV VLPs were analyzed for capacity binding with the Histo-Blood Group Antigen (HBGA). The results showed a binding pattern comparable to the original Norovirus. The morphology and size of the NoV VLPs were also studied using transmission electron microscopy and dynamic light scattering, confirming fully formed particles, with an average size of 40 nm. The particles also contain two multicloning sites inserted in the sequence of two external loops that could be used for antigen presentation. These production and purification systems described can be used for large-scale production of Norovirus *virus-like particles* as vaccine platform candidates.

## Materials and methods

### Plasmid construction and organism

The coding region for the NoV VP1 protein (GenBank acc. Number: AF080551) was received in the plasmid pJ912 from *DNA2.0 *(Menlo Park, CA, USA), and codon optimized for expression in *P. pastoris*. Two multicloning sites were inserted in the sequence to facilitate cloning into the two external loops of the NoV VLPs. The first multicloning site includes SpeI, NarI and FspI restriction sites, and the amino acid sequence corresponds with TSGGAGSAH, inserted between A_296_ and T_306_ of the VP1 sequence. The second Multicloning site includes PvuI, NotI and ScaI restriction sites, and the amino acid sequence corresponds with RSGGGRGST, inserted between S_382_ and D_392_ of VP1 sequence. The plasmid also contains the α-factor secretion signal, and the gene for resistance to zeocin. The coding region of the NoV VP1 protein was cloned after the coding region for the α-factor and the expression was regulated under the methanol inducible AOX1 promoter. *P. pastoris Bg11* (HIS^+^, Mut^s^) was provided by *Biogrammatics* (Carlsbad, CA, USA) and transformed by electroporation with 10 μg of linear plasmid [44].

*P. pastoris* transformants were grown on Yeast Extract Peptone Dextrose Medium (YPDS) plates containing: yeast extract 1% (w/v), peptone 2% (w/v), dextrose 2% (w/v), sorbitol 1 M, agar 2% (w/v) and zeocin 400 μg/mL and stored at 4°C. For long-term storage, *P. pastoris* strains were stored at −80°C [45,46].

### Protein expression

Eight independent *P. pastoris* clones were tested for VLP secretion. After growth at 30°C for 24 h in 50 mL of buffered medium with glycerol for yeast (BMGY, [100 mM potassium phosphate, pH 6.0, 1.34% (w/v) YNB, 4×10^−5^% (w/v) biotin, and 1% (v/v) glycerol]), cells were harvested by centrifugation and resuspended in 25 mL of buffered medium with methanol for yeast (BMMY, [100 mM potassium phosphate, pH 6.0, 1.34% (w/v) YNB, 4×10^−5^% (w/v) biotin, and 0.5% (v/v) methanol]). The cultures were maintained at 30°C during 120 h with vigorous agitation and induced with methanol every 24 h to a final concentration of 0.5% (v/v). The secreted protein in the supernatant was analyzed by 0.1% (w/v) sodium dodecyl sulfate (SDS)-15% (w/v) polyacrylamide gel electrophoresis (PAGE) and western blot. For the western blot, the proteins ran on an SDS-PAGE gel and were transferred onto a PDVF membrane (Millipore) and incubated at 37°C for 1 h in PBS with 5% non-fat dry milk. Subsequently, a rabbit anti-NoV serum was used for immunological identification of the VP1 protein, and incubated for 2 h at 25°C with agitation at a ratio of 1:2000. The membrane was washed with PBS-Tween-20 0.5% for 20 min and incubated for 1 h with GAR-HRP (1:3000) (BioRad). Color was developed using DAB (diaminobenzidine, Sigma), following the product instructions. The colony with the highest relative expression was selected for large-scale expression in the bioreactor.

### Fermentation

Fermentation was carried out as described previously [33,47]. A single *P. pastoris* colony was grown in 5 mL of YPD [1% (w/v) yeast extract, 2% (w/v) peptone, and 2% (w/v) dextrose] overnight at 30°C. This culture was used to inoculate 100 mL of BMGY. The new culture was grown in a baffled flask at 30°C while shaking until it reached an OD_600_ of ~20. This culture was used to inoculate 1.5 L of fermentation medium.

For the fermentation a Bioflo 3000 (New Brunswick Scientific, 2.5 L working volume) was used, interfaced with AFS-Biocommand Bioprocessing software version 2.6 (New Brunswick Scientific, Enfield, CT, USA) for data acquisition and control. The fermentation media consisted of 1.5 L of modified basal salts medium [0.23 gL^−1^CaSO_4_ *·* 2H_2_O, 4.55 gL^−1^ K_2_SO_4_, 3.73 gL^−1^ MgSO_4_ · 7H_2_O, 1.03 gL^−1^ KOH, and 6.68 mL L^−1^ H_3_PO_4_, 5% (v/v) glycerol] and 0.5 mL Antifoam 204 (Sigma, St. Louis, MO). For pH control, ammonium hydroxide [15% (v/v)] and phosphoric acid [15% (v/v)] were used. PTM_1_ trace salts (24 mM CuSO_4_, 0.53 mM NaCI, 19.87 mM MnSO_4_, 0.83 mM Na_2_MoO_4_, 0.32 mM boric acid, 2.1 mM CoCl_2_, 0.15 mM ZnCl_2_, 0.23 M FeSO_4_, and 0.82 mM biotin) were aseptically added at 4.35 mL L^−1^ after sterilization prior to inoculation. Temperature and pH were maintained at 30°C and 6, respectively. Dissolved oxygen (DO) was maintained at 40% of saturation and controlled by a DO-cascade agitation (with a maximum speed of 1000 rpm), supplemented with pure oxygen when needed, and measured with an InPro6110/220 electrode (Mettler-Toledo GmbH, Germany).

The batch phase was maintained until all the glycerol was depleted, as indicated by a sharp rise in DO (DO spike). At that point the culture was fed with methanol (100% methanol, with 12 mL PTM_1_ L^−1^) and maintained at a constant concentration of 2 g/L. Every 24 hours, samples were collected and centrifuged in order to separate the culture supernatant from the pellet. The dry cell weight (DCW) was measured and the culture supernatant was kept for further analysis.

### Protein purification

After the fermentation the culture was centrifuged at 4500 g at 4°C for 30 min to separate the supernatant from the pellet. The supernatant was filtered and dialyzed against 25 mM potassium phosphate, pH 8.0, for anion-exchange chromatography. The chromatography was performed using a HiTrap Q XL 5 mL column (Amersham Biosciences, Pittsburgh, PA, USA) and an AKTA Explorer (Amersham Biosciences, Pittsburgh, PA, USA). The column was equilibrated with 25 mM potassium phosphate, pH 8.0, and after loading 50 mL of supernatant, the column was washed with the same buffer. NoV VLPs were eluted using a gradient of 25 mM potassium phosphate, pH 8.0, 1 M NaCl. The chromatography fractions containing NoV VLPs were stored at 4°C.

### Cell lysis

Cell lysis was assayed through detection of α-glucosidase using pNPG as described in literature [47-49]. A 4 mg/mL solution of pNPG at was mixed in BSG (gelatin 0.1% w/v, NaCl 0.85% w/v, KH_2_PO_4_ 0.03% and Na_2_HPO_4_ 0.06% w/v) and aliquots of different volumes were taken from the culture. After centrifugation at 4500 g at 4°C for 10 min, the cell pellet was separated from the supernatant and lysed following the protocol described in *EasySelect Pichia Expression Kit* (Life technologies*, Grands Island, NY*). The lysates were mixed with BSG and 500 μl aliquots were placed in 1.5 mL microfuge tubes containing 100 μl of pNPG at 4 mg/mL. These tubes were incubated at 37°C for 30 minutes, after which the reaction was stopped by the addition of 250 μl of 1 M Na_2_CO_3_. Finally, the absorbance was measured at 405 nm. An aliquot from the fermentation was centrifuged under the same conditions as described above, and the supernatant was separated from the pellet and filtered by 0.22 μm to eliminate any cellular debris. The same protocol was performed on the fermentation sample and OD at 405 nm was measured. All the negative controls were developed with the assay and the background was subtracted from the results. Each point represents the average of three independent experiments.

### Particle size analysis

The size of the NoV VLPs was analyzed with *dynamic light scattering* (DLS). Suspensions of the NoV VLPs were prepared using 25 mM potassium phosphate buffer, pH 8.0, as dispersant and then transferred to 400 μL disposable sizing cuvettes. DLS measures the diffusion of the particles moving under Brownian motion using a Zetasizer Nano-ZS (Malvern Instruments Ltd, Worcestershire, UK). The results were calculated as the average of six consecutive measurements recorded at 25°C. Pre-determined viscosity and refractive index values were used in particle size calculations. The intensity and volume counts were plotted against particle size for each of the virus particle suspensions.

### Transmission Electronic Microscopy (TEM)

Purified NoV VLPs were loaded onto copper mesh grids (Electron Microscopy Sciences, Fort Washington, PA) and stained with 1.5% Uranyl Acetate. The samples were imaged using a F20 FEI Technai 200 kV field transmission electron microscope at the Cornell Center for Materials Research. Images were taken with a Gatan Orius dual-scan CCD camera at 1–3 s exposure time.

### Saliva-VLP binding assay

A 5 mL sample of human saliva phenotype A was diluted 1:1000 with PBS, boiled at 100°C for 5 min to denature potential anti-NoV antibodies and centrifuged at 7000 g for 10 min. From the supernatant, 100 μl/well were used to coat a 96-well microtiter plate, which incubated overnight at room temperature. NoV VLPs at different concentrations were added to the wells and incubated for 2 h at room temperature, after which the plate was blocked with 5% non-fat dry milk in PBS for 2 h at room temperature. The NoV VLPs were detected using an antiNoV serum (1:1000) for 1.5 h at room temperature, followed by GAR-HRP (1:3000) (BioRad) for another hour at room temperature. Color was developed using TMB Substrate Kit (Thermo Scientific), and the OD was measured at 450 nm using a Tecan GENios. Six independent experiments were conducted for each concentration point, and the highest VLP concentration was used for secondary control without saliva. Other secondary controls with the secondary antibodies were also carried out using the assay. The data are presented as the average of the results obtained for each well after background subtraction.

## Abbreviations

DLS: Dynamic light scattering
GAR: Goat anti-rabbit
HBGA: Histo-Blood Group Antigens
NoV: *Norovirus*
PBS: Phosphate buffered saline
PNPG: P-nitrophenyl-β-D-glucopyranoside
TEM: Transmission electronic microscopy
VLPs: *Virus-like particles*
